# Reducing Preschool Exclusionary Discipline Practices Through Infant and Early Childhood Mental Health Consultation: Findings from the Jump Start Program

**DOI:** 10.3390/children13030328

**Published:** 2026-02-26

**Authors:** Yaray Agosto, Morgan D. Darabi, Ana Robleto, Maite Schenker, Bianca Caceres, Elizabeth Erban, Tania Ramirez, Rachel Spector, Ruby Natale

**Affiliations:** 1 Mailman Center for Child Development, University of Miami Miller School of Medicine, Miami, FL 33136, USA; yagosto@med.miami.edu (Y.A.); mdarabi@miami.edu (M.D.D.); arobleto@med.miami.edu (A.R.); mxs2515@med.miami.edu (M.S.); 2 Community Health of South Florida, Inc., Miami, FL 33190, USA; bcaceres@chisouthfl.org; 3 Family Central, Inc., Miami Shores, FL 33138, USA; elizabetherban@familycentral.org; 4 Clinical Services Department, Jewish Community Services of South Florida, Miami, FL 33181, USA; tramirez@jcsfl.org; 5 The Children’s Trust of Miami-Dade County, Miami, FL 33129, USA; rachel@thechildrenstrust.org

**Keywords:** infant and early childhood mental health consultation, early care and education, exclusionary discipline practices, suspensions, expulsions, soft expulsions

## Abstract

**Highlights:**

**What are the main findings?**

- Childcare centers reported significant reductions in both traditional suspensions/expulsions and soft expulsions after participation in Jump Start, an infant and early childhood mental health consultation model.
- Jump Start was associated with reductions in soft expulsions when implementation significantly improved childcare center discipline policies.

**What are the implications of the main findings?**

- Given reductions in traditional suspensions/expulsions and negative developmental trajectories associated with exclusionary discipline practices, researchers, educators, and policy makers should increasingly focus their efforts on the reduction in soft expulsions.
- When aiming to reduce soft expulsions, educators and policy makers should select interventions that support childcare centers in improving their policies related to exclusionary discipline practices.

**Abstract:**

Background/Objectives: National data show that about 250 preschoolers are suspended or expelled daily in the United States. Jump Start is a multi-tiered infant and early childhood mental health consultation program that strengthens early care and education centers’ capacity to support children’s social–emotional development and prevent school suspension and expulsion. This retrospective study examined center-level exclusionary discipline practices, Jump Start participation, and related changes in discipline and expulsion policies. Methods: Data from 270 early care and education centers across Miami-Dade County that received Jump Start services during one of three academic years (2022–2023, 2023–2024, or 2024–2025) were included. Analyses examined associations between baseline exclusionary discipline practices, program duration, discipline and expulsion policy changes, and post-Jump Start exclusionary discipline practices. Results: Statistically significant reductions were observed in the frequency of traditional suspensions/expulsions and soft expulsions following Jump Start participation. The association between the Jump Start duration and post-Jump Start soft expulsions was significantly moderated by changes in center discipline policies, such that Jump Start was effective at reducing soft expulsions only when discipline policies showed meaningful improvement. Conclusions: Infant and early childhood consultation models, such as Jump Start, show promise in reducing exclusionary discipline practices, especially when implementation improves discipline policies.

## 1. Introduction

National data indicate that approximately 250 preschool children are suspended or expelled from classrooms each day in the United States [1]. These exclusionary discipline practices have been recognized as an ongoing problem in early care and education (ECE) settings and occur at rates exceeding those observed in K-12 education, with preschoolers expelled more than three times more than older students [2,3]. More specifically, Hispanic and Black preschool boys account for 66% of suspension and expulsion incidents, despite comprising only 46% of enrollment [3,4]. Exclusionary discipline in early childhood is associated with a range of adverse outcomes across life, including later school suspension and expulsion, academic failure, school dropout, and increased risk of justice system involvement, which collectively contribute to the “preschool to prison pipeline” and widen existing racial and socioeconomic inequities [2,5].

Beyond traditional suspensions and expulsions, ECE settings frequently rely on soft expulsion, an informal and often undocumented form of exclusionary discipline [6,7]. Soft expulsion refers to practices that implicitly push children out of the childcare center without formally mandating removal, particularly in contexts where expulsion is discouraged or prohibited [6]. Common examples include repeatedly sending children home early, removing children from their regular educational environment, reducing attendance hours, frequently contacting caregivers to pick up a child, or suggesting that a program is not a “good fit,” ultimately leaving families with little choice but to withdraw their child [6]. Because these practices are rarely captured in administrative data, the prevalence of exclusionary discipline practices in early childhood is likely underestimated, obscuring the full scope of the problem [7]. Importantly, evidence suggests that soft expulsion persists even in programs with explicit non-expulsion policies, often reflecting limited staff capacity, insufficient behavioral supports, and beliefs that some children’s behaviors cannot be effectively managed within the existing classroom structures [6].

Infant and early childhood mental health consultation (IECMHC) is an evidence-based approach that promotes children’s social–emotional development and helps to reduce suspension and expulsion rates in ECE settings [8,9,10]. IECMHC utilizes a multifaceted approach (program-, classroom-, and child-level consultations) in which mental health consultants (MHC) build ECE directors’ and classroom teachers’ capacity to effectively address children’s social and emotional needs through positive and supportive interactions in ECE settings [8,11]. Rather than providing direct therapeutic services to children, IECMHC focuses on strengthening adult skills, relationships, and systems that support young children’s social–emotional and behavioral health [12,13]. At its core, IECMHC is designed to facilitate meaningful shifts in early childhood professionals’ beliefs, attitudes, and practices, with increasing emphasis on equity-informed approaches that encourage reflection on bias and promote caregiving practices that are attentive to race, gender, language, and class [5,11].

Building on the Georgetown Framework for IECMHC [14], the Jump Start (JS) Early Childhood Consultation program is a multi-tiered IECMHC model operating at the program, classroom, and child levels. It is delivered by trained MHCs to strengthen ECE centers’ capacity to support children with challenging behaviors and to enhance behavior management practices before issues escalate to traditional suspension/expulsion or soft expulsion. JS places explicit emphasis on center-wide discipline and expulsion policies, with the goal of reducing reliance on exclusionary discipline practices and promoting developmentally appropriate, equity-informed approaches to support behavior. The Georgetown IECMHC framework posits that consultation may influence exclusionary discipline practices both through classroom-level behavior management and through shifts in center-wide policies [14]. Changes in discipline and expulsion policies specifically may influence the strength of the association between JS program duration and reductions in exclusionary practices. While IECMHC models have demonstrated effectiveness in reducing exclusionary discipline practices [9,15,16], limited research has examined associations between changes in center-level policies and both traditional suspensions/expulsions and soft expulsions. Therefore, the purpose of the present study was to examine: (1) the relationships between center-level exclusionary discipline practices, JS participation, and related changes in discipline and expulsion policies; (2) changes in traditional suspensions/expulsions and soft expulsions from baseline to post-program assessment; and (3) whether changes in discipline and expulsion policies moderated the association between the JS participation duration and post-program exclusionary discipline practices.

## 2. Materials and Methods

### 2.1. Participants

This retrospective analysis included 270 ECE centers across Miami-Dade County that received an average of 7 months of JS services during the 2022–2023, 2023–2024, or 2024–2025 academic years. ECE programs were identified and recruited from a list provided by the funding agency, The Children’s Trust. Total center enrollment ranged from 3 to 288 children, with a total of 16,021 children across all centers. On average, each center included 61 children, 5 classrooms, and 11 staff members. Children were racially (61% White, 21% Black, 12% multiracial) and ethnically diverse (69% Hispanic and 3% Haitian), and gender was evenly distributed (49% female). On average, center directors reported that 11% of children at their center had special needs. Over one third of children were enrolled in a school readiness or childcare subsidy program (36%), indicating lower socioeconomic status, and more than half of the ECE centers were enrolled in the county’s Thrive by Five Initiative, a local program designed to improve access to high-quality ECE centers for children in high-poverty areas [17]. The University of Miami’s Institutional Review Board approved this retrospective review of JS data to assess the program’s effectiveness and inform future program improvements (IRB study number 20251177).

### 2.2. Measures

#### 2.2.1. Demographics

During the enrollment phase, center directors completed both an intake form and a program demographics form, which collected information about their professional background, ECE center characteristics, and the children served.

#### 2.2.2. Frequency of Exclusionary Discipline

Incidences of exclusionary discipline practices were assessed via director self-report at baseline and immediately after completing JS using the Health Environment Rating Scale–Program (HERS-P) Self-Assessment. MHCs were trained to follow standardized instructions when administering the measure, to ensure consistent interpretation of items across participating directors. Respondents reported the number of traditional suspensions/expulsions that occurred at their center in the last 12 months, which was consistent with how formal exclusionary incidents are typically tracked and reported at the program level [2,3]. To assess the frequency of soft expulsions, directors were asked: “On average, how many times during the week are children that exhibit challenging behaviors removed from a classroom and sent to the office or another classroom?” This behavioral indicator is consistent with prior research operationalizing soft expulsion through reports of classroom removal practices [7], and weekly frequency was used because such practices are informal and not systematically documented in administrative records [6,7]. Because the JS duration averaged approximately 7 months, the differing time frames were intended to align with standard annual documentation of traditional suspensions/expulsions, while capturing more proximal changes in weekly classroom practices related to soft expulsions.

#### 2.2.3. Discipline and Exclusion Policies

The Health Environment Rating Scale–Program (HERS-P) is a 30 min interview tool developed by the study team that examines the adoption of center-wide policies related to the JS model. Similar to the team’s Health Environment Rating Scale–Classroom observation measure [18], the HERS-P aligns with core national standards, specifically those set by the National Association for the Education of Young Children [19] and Caring for Our Children [20], and covers four domains: safety, trauma-informed behavioral supports, self-care, and communication. Interviews were conducted with center directors by MHCs who completed a 5-h HERS-P training and achieved at least 80% agreement on ratings of recorded HERS videos to establish interrater reliability. For the purposes of this study, analyses focused on the total scores from two subdomains within behavioral supports: (1) expulsion and suspension policy (e.g., “The program has an expulsion and suspension policy that eliminates or severely limits expulsion, suspension, or other exclusionary disciplines”) and (2) discipline policy (e.g., “The program has established developmentally appropriate social–emotional discipline practices and intervention procedures”). The HERS-P is scored on a 7-point Likert scale ranging from “far below standards” (1) to “exceeds standards” (7). Both subscales of the HERS-P exhibited acceptable internal reliability (Suspension and Expulsion Policy: α = 0.81–0.87; Discipline Policy: α = 0.71–0.82).

### 2.3. Procedures

The JS program is a multi-tiered IECMHC model implemented in ECE centers across Miami-Dade County. To determine readiness for collaborative consultation, centers were initially screened for recent participation in formal preschool expulsion-reduction initiatives. In addition, MHCs conducted semi-structured interviews with center directors prior to enrollment that covered program leadership, discipline practices, and capacity to engage in reflective consultation and programmatic change. Centers were considered ready for participation when directors indicated no prior participation in expulsion-reduction initiatives and demonstrated openness to partnership and willingness to engage in consultation activities, including completing required assessments and allowing MHC access to staff and classrooms for observation. Demographic data were collected from centers that met readiness criteria and enrolled in the program. Next, center directors and selected classroom teachers completed informed consent and demographic questionnaires. Two teachers per center were chosen by the center director to also collaborate with the MHC. Selections were made based on the teachers’ needs for growth, classroom challenges, and their own motivation to participate.

JS services were offered for up to 10 months per center. MHCs were assigned caseloads of approximately four centers and spent one full day per week at each site. Consultation activities included program-level consultation, with administrators focused on policies, practices, and exclusionary discipline prevention strategies (approximately two hours per visit); classroom-level consultation focused on supporting teachers’ capacity to promote children’s social–emotional development and address challenging behaviors using developmentally appropriate strategies (approximately four hours per visit); and child-level consultation with caregivers and teachers of identified children with persistent behavioral challenges (that placed them at risk for traditional suspension/expulsion or soft expulsion) to help them reflect on the meaning of behaviors and develop individualized strategies to strengthen the protective factors (approximately two hours per visit). Two child-level cases per center were selected by directors and teachers with parents’ consent.

Consultant caseload assignment was guided by cultural and language compatibility whenever possible. Centers reported the primary language(s) spoken by staff and the racial and ethnic composition of teachers and administrators at intake. Services were provided in English, Spanish, and Haitian Creole, and consultants were matched to centers based on shared language, cultural background, or prior professional relationships to facilitate engagement and implementation.

### 2.4. Analysis

Descriptive and correlational analyses explored relationships among exclusionary discipline practices at baseline, JS program duration, discipline and expulsion policy changes, and exclusionary discipline practices after completing JS. Two Wilcoxon signed-rank tests were conducted to evaluate pre- and post-JS differences in traditional suspensions/expulsions and soft expulsions. In addition, four moderation analyses were conducted using the PROCESS macro with 5000 bootstrap samples to examine whether the relationships between JS program duration and post-program exclusionary discipline practices (traditional suspensions/expulsions and soft expulsions) were moderated by changes in ECE center policies (discipline and expulsion policies). Total center enrollment and frequency of exclusionary discipline practices prior to JS were included as covariates in each model.

## 3. Results

### 3.1. Preliminary Analyses

At baseline, center directors reported an average of 0.25 traditional suspensions/expulsions in the last 12 months, with 16% of centers reporting at least one traditional suspension/expulsion in the last 12 months. In addition, center directors reported an average of 2.22 soft expulsions per week, with 45% of centers reporting at least one soft expulsion per week. On average, ECE centers implemented JS for 7 months between baseline and post-program assessment. During this time, center directors reported positive changes in their discipline (M = 1.06) and expulsion (M = 0.95) policies. Significant associations emerged between changes in discipline and expulsion policies (*r* = 0.49, *p* < 0.001). After completing JS, center directors reported an average of 0.13 traditional suspensions/expulsions in the last 12 months, with 11% of ECE centers reporting at least one traditional suspension/expulsion in the last 12 months. In addition, center directors reported an average of 0.58 soft expulsions per week, with 33% of ECE centers reporting at least one soft expulsion per week. Significant associations emerged between traditional suspensions/expulsions and soft expulsions after completing JS (*r* = 0.15, *p* = 0.02). See Table 1.

### 3.2. Effect of Jump Start on Exclusionary Discipline Practices

A first Wilcoxon signed-rank test was conducted to evaluate the effect of JS on the frequency of traditional suspensions/expulsions. Results indicated a statistically significant difference in the frequency of traditional suspensions/expulsions before and after JS, *Z* = −2.56, *p* = 0.01. Traditional suspensions/expulsions decreased from 0.25 (Md < 1) to 0.13 (Md < 1) in the last 12 months, suggesting that JS significantly reduced traditional suspensions/expulsions.

A second Wilcoxon signed-rank test was conducted to evaluate the effect of JS on the frequency of soft expulsions. The results indicated a statistically significant difference in the frequency of soft expulsions before and after JS, *Z* = −4.79, *p* < 0.001. Soft expulsions decreased from 2.22 (Md < 1) to 0.58 (Md < 1) per week, suggesting that JS significantly reduced soft expulsions.

### 3.3. Moderation of Jump Start on Exclusionary Discipline Practices by Policy Changes

**Figure 1:**
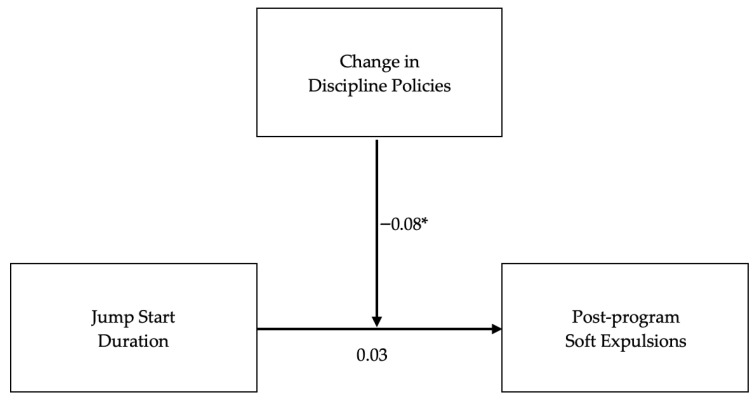
Moderation of duration on soft expulsions by discipline policy change. *Note*. Center enrollment and soft expulsions at baseline included as covariates. * *p* < 0.05.

The overall model examining the moderation of JS program duration and soft expulsions after completing JS by changes in discipline policies, controlling for total center enrollment and soft expulsions at baseline, was statistically significant, *R^2^* = 0.05, *F*(5, 240) = 2.63, *p* = 0.02. A significant interaction emerged between the JS program duration and changes in discipline policies, *b* = −0.08, *SE* = 0.04, *t*(240) = −1.98, *p* < 0.05. This interaction accounted for a significant portion of the variance in soft expulsions, Δ*R*^2^ = 0.02, *F*(1, 240) = 3.93, *p* < 0.05. See Figure 1.

**Figure 2:**
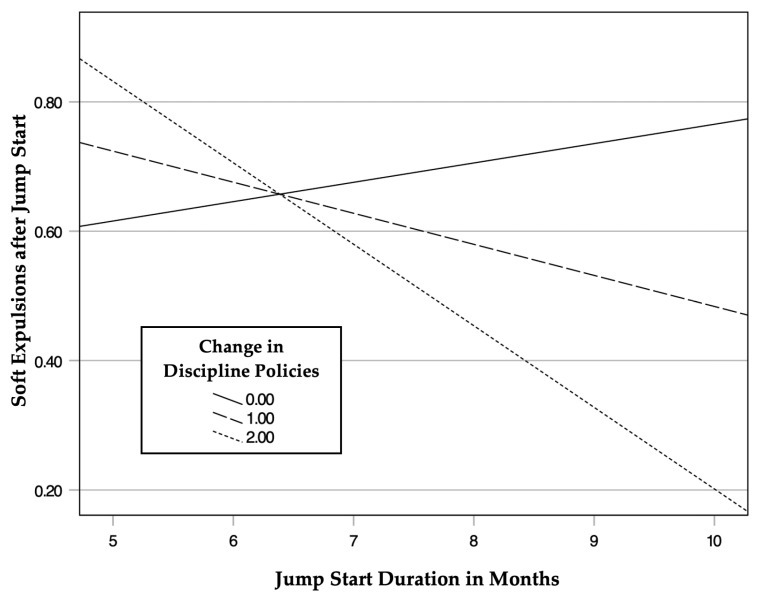
Conditional effects of Jump Start duration on soft expulsions at different levels of discipline policy change. Note. Center enrollment and soft expulsions at baseline included as covariates.

Simple slopes were analyzed to probe this interaction at three levels of discipline policy change (16th, 50th, and 84th percentiles). When there was no change to discipline policies (ΔPolicy = 0), the JS program duration did not significantly predict post-program soft expulsions (*b* = 0.03, *p* = 0.58). Similarly, at low levels of policy change (ΔPolicy = 1), the effect remained non-significant (*b* = −0.05, *p* = 0.17). However, when changes to discipline policies were high (ΔPolicy = 2), a longer JS program duration was significantly associated with lower post-program soft expulsions (*b* = −0.13, *SE* = 0.05, *t*(240) = −2.47, *p* = 0.01). These results suggest that JS is effective at reducing soft expulsions when implementation significantly improves center discipline policies. See Table 2 and Figure 2.

The three models examining JS program duration and soft expulsions by changes in expulsion policies, JS program duration and traditional suspensions/expulsions by changes in discipline policies, and JS program duration and traditional suspensions/expulsions by changes in expulsion policies did not include significant moderation effects (all *p* > 0.12).

## 4. Discussion

The current study examined whether participation in JS, a multi-tiered IECMHC model delivered at the program, classroom, and child levels, was associated with changes in exclusionary discipline practices within ECE centers. Although JS includes consultation activities across multiple levels, the current analyses focused specifically on center-level outcomes, including traditional suspensions/expulsions and soft expulsions, as well as changes in center-wide discipline and expulsion policies. In particular, this study explored how changes in ECE center policies influenced the relationship between the JS program duration and exclusionary discipline practices after completing JS. Overall, findings suggest that participation in JS was associated with meaningful reductions in exclusionary discipline practices, with the most robust and consistent associations observed for soft expulsion practices.

Consistent with prior research demonstrating that IECMHC models are effective in reducing suspension and expulsion in early childhood settings, participating centers reported relatively low rates of traditional suspensions/expulsions at baseline, with modest changes observed following JS participation [9,15,16]. Much of this prior work, including studies by Gilliam et al., has focused solely on traditional suspensions and expulsions, which are increasingly rare in jurisdictions with strong policy guidance discouraging these practices [2,5,15]. In the present study, soft expulsion practices were substantially more prevalent at baseline than traditional suspension/expulsion practices and demonstrated significant reductions at post-program assessment. Nearly half of participating ECE centers reported engaging in at least one soft expulsion per week at baseline, whereas only one-third reported any soft expulsions following completion of JS. These findings align with emerging evidence suggesting that soft expulsion practices are both more prevalent and potentially more harmful to children’s continuity of care and social–emotional development than traditional exclusionary discipline practices, particularly because it operates outside formal reporting and accountability mechanisms. Together, these results suggest that soft expulsion may represent a more sensitive and prevalent indicator of exclusionary discipline practices in contexts where formal suspension and expulsion are discouraged or restricted.

The observed reductions in soft expulsion are especially notable, given the growing evidence that such practices often replace traditional exclusionary discipline practices despite explicit non-expulsion policies [7]. Soft expulsion practices are often undocumented and therefore remain largely underreported in administrative data, despite their potential to disrupt children’s continuity of care and disproportionately affect children from historically marginalized backgrounds. The present findings underscore the importance of explicitly measuring soft expulsion when evaluating efforts to reduce exclusionary discipline practices in ECE settings and suggest that program-level consultation may play a critical role in addressing these less visible practices.

Moderation analyses further indicated that reductions in soft expulsion were strongest within ECE centers that demonstrated meaningful improvements in discipline policies during participation in JS. Specifically, longer engagement in JS was associated with fewer soft expulsions only when centers exhibited improved discipline policies. In contrast, no such associations were observed among centers with minimal or no policy change. This pattern suggests that multi-tiered consultation efforts may be most effective at reducing informal exclusionary discipline practices when classroom- and child-level supports are reinforced by clear, developmentally appropriate, and equity-informed center-level policies. No significant moderation effects were observed for traditional suspensions/expulsions. This likely reflects the low rates of traditional suspension/expulsion observed at baseline, which may have limited variability and statistical power to detect moderation effects. Together, these findings suggest that while IECMHC models may contribute to maintaining low levels of traditional exclusionary discipline practices, their greatest impact may be in reducing soft expulsion practices that remain more common and less regulated.

### 4.1. Limitations

Several limitations should be considered when interpreting these findings. First, exclusionary discipline practices were self-reported by center directors and may be subject to recall bias or social desirability bias. Although a standardized self-assessment measure was used, some variability in how exclusionary discipline practices, particularly soft expulsion, were interpreted or categorized may remain, with potential implications for construct validity. In addition, HERS-P interviews were conducted by the same MHCs providing JS services, introducing the potential for expectancy bias.

Second, the baseline rates of traditional suspensions/expulsions were relatively low. This likely reflects the broader policy and practice context of Miami-Dade County, where coordinated, county-wide initiatives aimed at reducing preschool suspensions and expulsions have been in place for several years. Efforts led by the Early Learning Coalition and partnerships among local early childhood and mental health organizations may have contributed to the widespread adoption of non-expulsion policies prior to centers’ participation in JS [21]. These same broader system-level efforts should be considered when interpreting changes during the study period.

Another important consideration is the study’s retrospective design and lack of a control group, which limits the ability to draw causal inferences about program effects. To reduce potential confounding from prior intervention exposure, centers were screened prior to enrollment for recent participation in formal preschool expulsion-reduction initiatives, and those with intensive prior involvement were not eligible. This step was intended to strengthen the internal validity and minimize contamination from previous targeted supports. As such, observed improvements should be interpreted as associations occurring during JS implementation, rather than definitive evidence of the program effects. Lastly, overall participation in JS may have influenced center policies, limiting conclusions regarding the temporal ordering of program duration, policy changes, and reductions in soft expulsions.

### 4.2. Future Directions

Despite these limitations, the present findings have important implications for policy and practice. The results suggest that IECMHC models that explicitly target program-level discipline policies may be particularly effective in reducing soft expulsions, an exclusionary discipline practice that is often underreported and difficult to track. Interventions that focus solely on reducing traditional suspensions and expulsions may underestimate the true scope of exclusionary discipline practices and overlook opportunities for meaningful change.

Future research should continue to refine the measurement of soft expulsion and explore how policy implementation fidelity, rather than policy presence alone, shapes exclusionary discipline practices. In addition, further research is needed to examine how center-level policy changes interact with classroom- and child-level consultation processes to influence exclusionary discipline practices over time and whether reductions in soft expulsions narrow disparities across centers serving historically marginalized populations. Lastly, researchers should explore policy changes as potential mediators between program participation and exclusionary discipline practices to further clarify the conceptual pathways underlying soft expulsions.

## 5. Conclusions

This study found that participation in JS may have been associated with reductions in exclusionary discipline practices at the center level, with the largest changes observed in soft expulsions. Although traditional suspensions and expulsions were relatively uncommon, soft expulsions were prevalent at baseline and occurred less frequently following program participation. Reductions in soft expulsion were observed primarily among centers that demonstrated meaningful improvements in discipline policies, suggesting that policy change may play an important role in shaping informal exclusionary practices. Together, these findings highlight the value of examining soft expulsion as a key indicator of exclusionary discipline practices and suggest that IECMHC models that attend to center-wide discipline policies may be well positioned to support more inclusive early care and education environments.

## Figures and Tables

**Table 1:** Descriptive statistics and associations between study variables.

	M	SD	1	2	3	4	5	6
*Baseline*								
1. Traditional Expulsions	0.25	0.78						
2. Soft Expulsions	2.22	13.76	−0.01					
*Jump Start*								
3. Duration in Months	7.10	2.32	0.01	−0.04				
4. Discipline Policy Change	1.06	0.88	0.05	−0.03	0.10			
5. Expulsion Policy Change	0.95	0.90	0.12	−0.06	0.13	0.49 **		
*Post-Program*								
6. Traditional Expulsions	0.13	0.38	0.25 **	0.00	−0.07	0.01	0.08	
7. Soft Expulsions	0.58	1.27	0.00	0.14 *	−0.12	−0.02	0.04	0.15 *

Note. Center enrollment at baseline included as covariate. ** *p* < 0.01. * *p* < 0.05.

**Table 2:** Conditional effects of Jump Start duration on post-program soft expulsions at different levels of discipline policy change.

Level of Policy Change	Effect (*b*)	*SE*	*t*	*p*	95% CI
Low (0)	0.03	0.05	0.55	0.58	[−0.08, 0.14]
Moderate (1)	−0.05	0.04	−1.37	0.17	[−0.12, 0.02]
High (2)	−0.13	0.05	−2.47	0.01	[−0.23, −0.03]

Note. CI = confidence interval. Center enrollment and soft expulsions at baseline included as covariates.

## Data Availability

Requests for data can be sent to the corresponding author.

## Abbreviations

The following abbreviations are used in this manuscript:

ECE	Early Care and Education
HERS-P	Health Environment Rating Scale-Program
IECMHC	Infant and Early Childhood Mental Health Consultation
IRB	Institutional Review Board
JS	Jump Start
MHC	Mental Health Consultant

## References

[1] New Data Reveal 250 Preschoolers Are Suspended or Expelled Every Day. https://www.americanprogress.org/article/new-data-reveal-250-preschoolers-suspended-expelled-every-day/.

[2] Gilliam W.S. (2005). Prekindergartners Left Behind: Expulsion Rates in State Prekindergarten Systems.

[3] Williams P.G., Yogman M. (2023). Addressing early education and child care expulsion. Pediatrics.

[4] Zeng S., Corr C.P., O’Grady C., Guan Y. (2019). Adverse childhood experiences and preschool suspension expulsion: A population study. Child Abus. Negl..

[5] Meek S.E., Gilliam W.S. (2016). Expulsion and Suspension in Early Education as Matters of Social Justice and Health Equity.

[6] Murphy K., Giordano K., Hoffstein-Rahmey D., Reizin R., Coyne A. (2024). Soft expulsion: A solution when there are no other options?. Child. Youth Serv. Rev..

[7] Steyer L., Gleit R., Hawkins C., Provençal M., Pearman F., Obradović J. (2025). Informal exclusionary discipline practices in US schools: Recent evidence and policy considerations. Soc. Policy Rep..

[8] Perry D.F., Allen M.D., Brennan E.M., Bradley J.R. (2010). The evidence base for mental health consultation in early childhood settings: A research synthesis addressing children’s behavioral outcomes. Early Educ. Dev..

[9] Silver H.C., Davis Schoch A.E., Loomis A.M., Park C.E., Zinsser K.M. (2023). Updating the evidence: A systematic review of a decade of Infant and Early Childhood Mental Health Consultation (IECMHC) research. Infant Ment. Health J..

[10] Trivedi P.A., deMonsabert J., Horen N.M. (2021). Infant and Early Childhood Mental Health Consultation: Overview of Research, Best Practices, and Examples.

[11] Duran F., Hepburn K., Irvine M., Kaufmann R.E., Anthony B., Horen N.M., Perry D.F. (2009). What Works? A Study of Effective Early Childhood Mental Health Consultation Programs.

[12] Center of Excellence for Infant and Early Childhood Mental Health Consultation Status of the Evidence for Infant and Early Childhood Mental Health Consultation. https://www.iecmhc.org/documents/CoE-Evidence-Synthesis.pdf.

[13] Natale R., Kolomeyer E., Futterer J., Mahmoud F.D., Schenker M., Robleto A., Horen N., Spector R. (2022). Infant and early childhood mental health consultation in a diverse metropolitan area. Infant Ment. Health J..

[14] Hunter A., Davis A., Perry D.F., Jones W. (2016). The Georgetown Model of Early Childhood Mental Health Consultation: For School-Based Settings.

[15] Gilliam W.S., Maupin A.N., Reyes C.R. (2016). Early childhood mental health consultation: Results of a statewide random-controlled evaluation. J. Am. Acad. Child Adolesc. Psychiatry.

[16] Newland R., Silver R.B., Herman R., Hartz K., Coyne A., Seifer R. (2024). Child-focused infant and early childhood mental health consultation: Shifting adult attributions to reduce the risk for preschool expulsion. Infant Ment. Health J..

[17] Thrive by Five. https://www.thechildrenstrust.org/about-us/what-we-fund/thrive-by-5/.

[18] Futterer J., Mullins C., Bulotsky-Shearer R.J., Guzmán E., Hidalgo T., Kolomeyer E., Howe E., Horen N., Sanders L.M., Natale R. (2024). Initial validation of the Health Environment Rating Scale–Early Childhood Consultation–Classroom (HERS-ECC-C). Infant Ment. Health J..

[19] National Association for the Education of Young Children Professional Standards and Competencies for Early Childhood Educators. https://www.naeyc.org/sites/default/files/globally-shared/downloads/PDFs/resources/position-statements/naeyc_standards_and_competencies_ps_final.pdf.

[20] American Academy of Pediatrics, American Public Health Association, National Resource Center for Health and Safety in Child Care and Early Education (2019). Caring for Our Children, National Health and Safety Performance Standards.

[21] Florida Department of Education, Office of Early Learning (2014). Position Statement on Expulsion and Suspension Prevention in Early Childhood Settings.

